# The Use of a Novel Container for Secured Transport and Storage of Biological Material for Quantitative Human RNA Analysis

**DOI:** 10.3390/ijms26010228

**Published:** 2024-12-30

**Authors:** Dorota Kostrzewa-Nowak, Alicja Trzeciak-Ryczek, Klaudyna Lewandowska, Thierry van de Wetering, Andrzej Ciechanowicz, Robert Nowak

**Affiliations:** 1 Department of Clinical and Molecular Biochemistry, Pomeranian Medical University in Szczecin, 72 Powstańców Wlkp. Al., 70-111 Szczecin, Poland; dorota.kostrzewa.nowak@pum.edu.pl (D.K.-N.); klaudyna.lewandowska@pum.edu.pl (K.L.); andrzej.ciechanowicz@pum.edu.pl (A.C.); 2 Institute of Biology, University of Szczecin, 13 Wąska St., 71-415 Szczecin, Poland; alicja.trzeciak-ryczek@usz.edu.pl; 3 The Centre for Molecular Biology and Biotechnology, University of Szczecin, 13 Wąska St., 71-415 Szczecin, Poland; 4 Department of Forensic Genetics, Pomeranian Medical University in Szczecin, 72 Powstańców Wlkp. Al., 70-111 Szczecin, Poland; thierry.vandewetering@pum.edu.pl; 5 Institute of Physical Culture Sciences, University of Szczecin, 17C Narutowicza St., 70-240 Szczecin, Poland; 6 Department of Pathology, Pomeranian Medical University in Szczecin, 1 Unii Lubelskiej St., 71-242 Szczecin, Poland

**Keywords:** digital PCR, leukocytes’ housekeeping genes, RNA quantitative analyses, samples’ transport

## Abstract

The transport of biological materials must protect samples from degradation and ensure courier safety. The main goal of this study was to evaluate the usefulness of a new type of container designed for the secured transport of biological material for storing samples for quantitative RNA analyses. This was achieved by analyzing changes in the expression of selected human leucocyte housekeeping genes (*ACTB*, *GAPDH*, and *Rack1*) using reverse transcription quantitative PCR (RT-qPCR) and digital PCR (RT-dPCR) techniques. Digital PCR analysis evidenced that the novel type of container retains a higher count of analyzed gene copies per µL of samples during 5 h of incubation time. The container ensures a low maintenance temperature for several hours, making it useful for sustaining the conditions for transporting biological samples. This novel container can be used to store and transport biological material to be analyzed by molecular techniques and can retain the stability of total RNA over several hours.

## 1. Introduction

Ribonucleic acid (RNA) isolation and purification techniques are well described and widely used in different research areas, including structural, biophysical, and biomedical studies [1,2]. With advances in scientific and diagnostic methods, RNA-based assays have become increasingly popular, raising interest in RNA molecules. Even though modern preparations provide large quantities of homogeneous, good-quality RNA, there are still issues with transport and the short- and long-term storage of RNA samples with regard to degradation.

Nucleic acid-based amplification tests are the modern golden standard of many diagnostic procedures and have increased in popularity for various research analyses. Therefore, molecular techniques that amplify nucleic acid molecules, including reverse transcription quantitative PCR (RT-qPCR), are commonly accepted as sensitive and specific [3]. Different biological materials (e.g., blood, urine, saliva, and serum) can be used in future investigations using molecular biology methods, such as RT-qPCR, Western blot, or ELISA [4,5,6,7,8]. Selecting the appropriate detection method is as critical as the safe and proper transportation and storage of biological material. Some molecular studies and medical diagnostic analyses require material transportation that protects the sample from degradation and ensures the safety of the courier.

It is well known that the appropriate storage of RNA samples is as critical as proper isolation and purification. Indeed, many factors, such as temperatures above 4 °C, oxygen access, and the presence of active nucleases that trigger RNA degradation, cause erroneous results [9,10]. Cryogenic storage in liquid nitrogen (−180 °C) is recommended for RNA samples [9], though it is not always possible, especially during sample transportation. Numerous commercial solutions help to avoid RNA degradation and transcription, including PAXgene Blood RNA tubes (PreAnalytiX, Qiagen/BD, Hombrechtikon, Switzerland), Tempus Blood RNA tubes (Applied Biosystems, Foster City, CA, USA), and RNAlater Stabilization Reagent (Thermo Fisher Scientific, Waltham, MA, USA) [11,12,13,14,15,16,17]. Although these special tubes help transport biological material for molecular analysis, they are still not sufficient to fulfil legal requirements for the transport of biological material that is potentially infectious. It is known that RNA samples could be shipped using dry ice preventing them from temperature degradation. However, high costs and logistical problems justify the search for new, more effective solutions that use not only lower temperature as a factor limiting RNA degradation but also prevent them from the availability of oxygen and hydration.

Providing safe transport of potentially infectious biological samples to medical diagnostic or molecular laboratories and preventing the degradation of the genetic material (especially RNA) will ensure accurate results. Guidance on the regulations for the transport of potentially infectious substances in the European Union requires the use of three containers: (i) primary packaging (e.g., a test tube or swab) containing the material to be tested, (ii) intermediate packaging (e.g., a hermetic waterproof container or plastic foil), and (iii) outer packaging (e.g., cardboard packaging or an envelope with a bubble insert) [18]. Additionally, polymers used for the production of laboratory consumables, in particular products to be used in medical laboratories, must meet very rigorous requirements and maintain their properties throughout the product’s life cycle. Among them, there are sterilization possibilities, biocompatibility, chemical resistance, durability, and mechanical resistance. It is worth noting that in packaging or bioengineering sectors, biodegradable biopolymers like polylactide (PLA) cannot be fully competitive with polymers from the category of commodity or engineering thermoplastics (e.g., polypropylene, PP; polyethylene terephthalate, PET; acrylonitrile butadiene styrene, ABS; etc.) [19]. Considering this, we decided to investigate the use of a final product that meets the abovementioned material requirements.

This paper presents a new type of container for the secured transport of biological material and describes its role in extending RNA sample quality. The novelty of this tool is its unique construction—the container consists of two separated layers, which makes it both secondary and external packaging, and is highly resistant to mechanical factors. Other advantages of the presented solution include (i) the optimization and ergonomics of the shape of the transporter, (ii) the possibility of modifying the absorption and cooling layer, and (iii) maintaining the correct and stable temperature during transport and sample description/registration at the laboratory, thanks to the presence of cooling and temperature-stabilizing elements during the transport. The primary goal of this study was to evaluate the usefulness of a new type of container designed for the secured transport of biological material for storing samples for quantitative RNA analyses based on changes in the expression of selected housekeeping genes using RT-qPCR and RT-dPCR techniques. The RT-dPCR technology was chosen as it is more advanced and sensitive than other PCR techniques and has been proposed for medical diagnostic molecular testing [20,21]. Additionally, we tested the temperature profile and mechanical resistance of the novel secured transport container.

## 2. Results

### 2.1. General Properties of the Container

**Figure 1:**
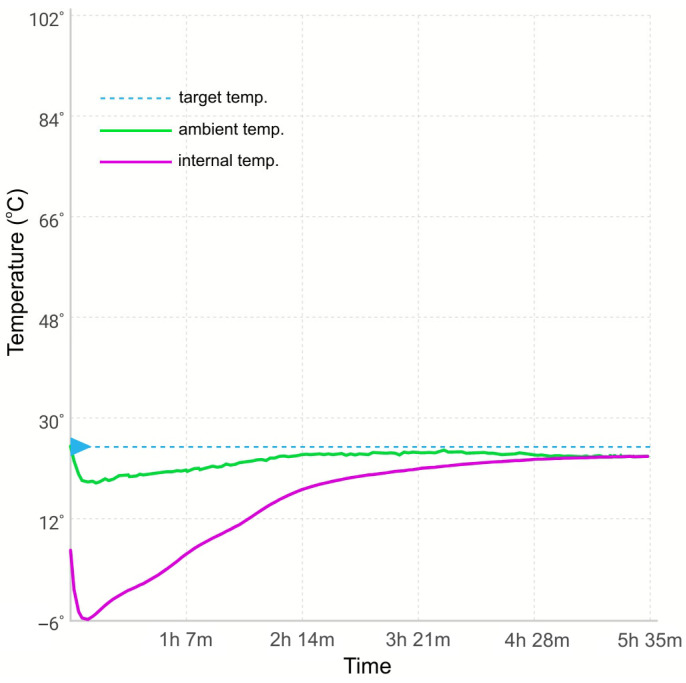
A representative example of the temperature profile of the container for secured transport of biological material. The blue dotted line indicates the target temperature value of +25 °C.

The transport of biological material, including RNA, requires not only the protection of such material from its degradation but also requires packaging characterized by mechanical, thermal, and chemical resistance. The development of prototypes is the stage of selecting appropriate materials for developing the production technology. Our previous research showed that PLA and PP maintain thermal conditions inside the prototype of the tested transporter similarly. On the other hand, ABS and PET maintained thermal conditions approximately 30–40 min shorter than PLA and PP (Supplementary Figure S1). Structural analysis and research at the prototype development stage became the reason for choosing PP as the final material. The results of the present study evidenced that the novel container did not lose its tightness after several freeze–thaw cycles. The container temperature profiles showed that the coolant placed between the walls of the container body maintained the reduced temperature for at least 2 hours (on average, approx. 2 h until reaching the temperature of +20 °C and approx. 5.5 h until reaching the temperature of +25 °C). The time for maintaining the reduced temperature depends on the temperature of the environment in which the container was placed. A representative example of the container’s temperature profile is shown in Figure 1.

### 2.2. Study Design

The RNA was extracted from human peripheral nuclear blood cells (white blood cells, WBCs), and the obtained solutions (RNA concentration equal to 41.49 ng/µL) were divided for evaluation into two probes named “control” and “test”, respectively. The control probes were stored at room temperature during the observation time, while the test probes were stored inside the cooled container (the initial container’s internal temperature was −5 °C ± 1 °C). The RNA concentrations were measured at several time points (0 min, at the beginning of the experiment, and then 15, 30, 60, 90, 120, 180, 240, and 300 min of incubation) in the case of the RNA integrity study and 0, 90, 120, and 300 min of incubation in the case of the RT-qPCR and RT-dPCR analyses. Additionally, at each time point, the quality of the RNA was estimated to assess whether the RNA was fully intact or how much it was degraded. RNA that is completely degraded has an RNA integrity number (RIN) of one, while fully intact RNA has a RIN value of 10. The stability of housekeeping genes for human WBC expression was assessed using RT-qPCR and the counts of RNA matrix for selected genes using RT-dPCR. The selection of time points in this study was based on the analysis of the container’s internal temperature profile. The housekeeping genes were selected from the most comprehensively characterized housekeeping genes for human leucocytes, namely, *ACTB* (β-actin), *GAPDH* (glyceraldehyde-3-phosphate dehydrogenase), and *Rack1* (receptor for activated C kinase 1) [22,23,24,25]. This study was replicated three times in the same laboratory conditions. The RNA samples were obtained from 3 to 4 different volunteers per trial. The blood samples were collected in 7.5 mL S-Monovette tubes with ethylenediaminetetraacetic acid (EDTA K3, 1.6 mg EDTA/mL blood) for future total RNA isolation (according to the protocol described in Section 4). The total RNA samples in each series were pooled in one tube and then divided into “control” and “test” probes. For each studied time point, three separated tubes of total RNA sample were prepared. After the incubation time, the RT-qPCR and RT-dPCR analyses were performed for each tube separately.

### 2.3. The Results of RNA Quantitative Analyses

The changes in the integrity of the studied RNA samples are presented in Table 1. It was found that during the incubation time, there were no significant changes in the RIN in the tested samples while the decrease in this parameter was noticed in the case of the control probes (Table 1).

**Figure 2:**
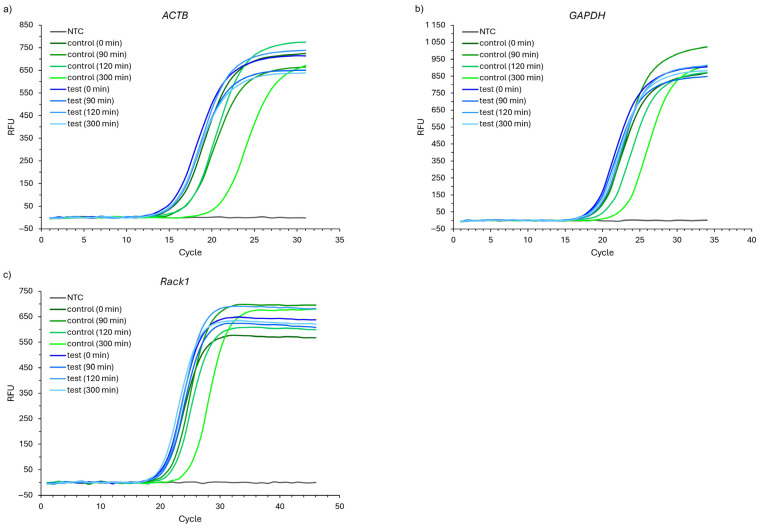
Representative results of the quantification amplification for (**a**) *ACTB*, (**b**) *GAPDH*, and (**c**) *Rack1* genes in analyzed samples in selected time points. NTC—no template control. Threshold values for the analyses equal the following: for *ACTB*—73.77; for *GAPDH*—80.79; and for *Rack1*—85.55, respectively. The figure presents one random series of the experiment.

**Figure 3:**
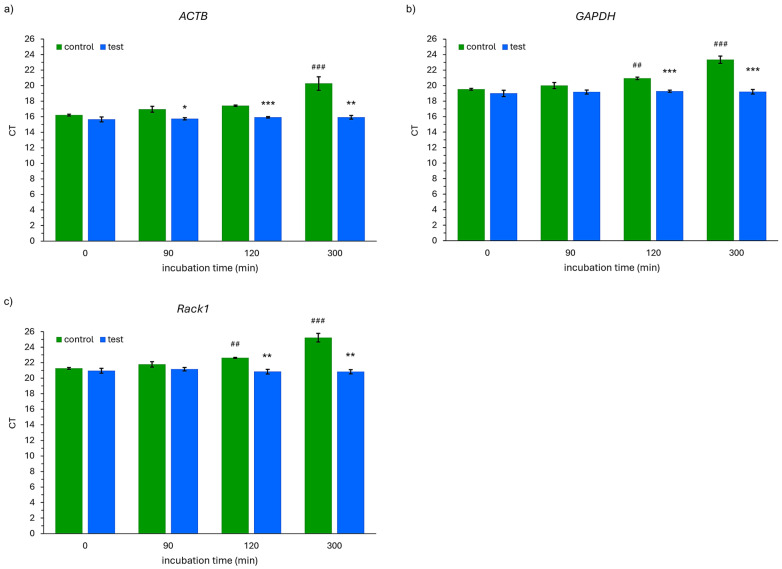
Obtained cycle threshold (CT) values for (**a**) *ACTB*, (**b**) *GAPDH*, and (**c**) *Rack1* genes in analyzed samples. * *p* < 0.05, ** *p* < 0.01, and *** *p* < 0.001 (control vs. test); ## *p* < 0.01 and ### *p* < 0.001 (compared to 0 min). The bars represent the mean and the whiskers represent the standard deviation of the triplicate experiment.

The results of the RT-qPCR analyses are presented in Figure 2 and Figure 3. The representative quantification amplification results for selected time points are presented in Figure 2, where one can observe the tendency to shift the cycle threshold (CT) between the control and test probes.

There were significant differences in the expression of the *ACTB*, *GADPH*, and *Rack1* genes between the control and test probes after 120 and 300 min of incubation (Figure 3). Also, a significant difference was observed after 90 min of incubation in the case of the *ACTB* gene (Figure 3a). The CT value obtained for the *ACTB* gene was significantly higher (*p* < 0.001) than the baseline (0 min) after 300 min of incubation of the samples outside the container (control probes, Figure 3a). It was observed that the CT values for the *GAPDH* gene were significantly higher after 120 (*p* = 0.009) and 300 (*p* < 0.001) minutes of incubation in comparison to the baseline values in the control probe (Figure 3b). Similarly, in the case of *Rack1* gene expression, significant differences in the CT values (as compared to 0 min) were observed after 120 (*p* = 0.007) and 300 (*p* < 0.001) minutes of incubation time (Figure 3c). There were no significant differences between the CT values at the analyzed time points in comparison to 0 min in the case of all the studied genes in the test probes.

**Figure 4:**
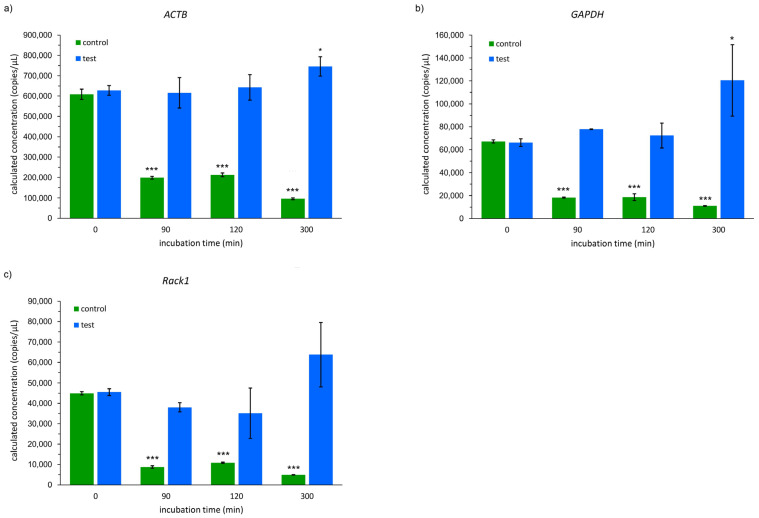
Calculated number of (**a**) *ACTB*, (**b**) *GAPDH*, and (**c**) *Rack1* gene copies (per µL) in analyzed samples obtained in RT-dPCR analyses. * *p* < 0.05 and *** *p* < 0.001 (compared to 0 min). The bars represent the mean and the whiskers represent the standard deviation of the triplicate experiment.

Additionally, an RT-dPCR analysis was performed due to being a more advanced and sensitive technique than qPCR. The representative results of the dPCR droplets of the analyzed genes are presented in the Supplementary Figure S2. In all the samples incubated in the container (test probes), the calculated copies of the *ACTB*, *GAPDH,* and *Rack1* genes per µL were significantly higher in comparison to the control probes (Figure 4). After 90 min of incubation, the number of copies was about 3.1-fold higher in the case of *ACTB* and about 4.3-fold higher in the case of the *GAPDH* and *Rack1* genes in the test probes over the control probes. More than three-fold higher values were found after 120 min of incubation in all the probes incubated in the storage containers (test probes) compared to the control probes. A 300 min long incubation of the samples resulted in an even bigger difference in favor of the container. It was observed that the number of *ACTB* gene copies per µL was about 7.8-fold higher than in the samples kept at room temperature. In the case of the *GAPDH* and *Rack1* genes, the difference was more than 10-fold (Figure 4). The control probes showed a significant reduction in analyzed gene copies as a function of the incubation time. Interestingly, when comparing a 300 min incubation with the baseline values of the test group, a significant increase in *ACTB* and *GAPDH* copies was observed.

## 3. Discussion

Secure and accurate storage of biological material during transport to medical diagnostic laboratories is of high importance with regards to avoiding pre-analytical errors and obtaining accurate results during laboratory testing. Plebani in his review points out that the most frequent laboratory errors are those made in the pre-analytical and post-analytical phase than during the test itself [26]. According to his work, the majority of errors are made before testing. There is increasing literature data confirming that most errors are identified in steps outside the laboratory [27,28]. On the other hand, proper material used for the production of consumables dedicated to the transport of biological material can have an important impact on decreasing its degradation and increasing the safety of users. The main body of the presented transporter is made from PP. Polypropylene is a lightweight polymer with a density of 0.90 g/cm^3^, making it suitable for many industrial applications. It has been proven to have excellent and desirable physical, mechanical, and thermal properties when used at room temperature. It is also relatively stiff and has a high melting point, low density, and relatively good impact resistance [29]. Polypropylene is resistant to many polar liquids, such as alcohols, organic acids, esters, and ketones [30,31]. The choice of PP as a polymer for the production of the container by injection molding technology was based on its ability to maintain the reduced temperature for the longest time among other materials with similar physicochemical properties and excellent chemical resistance to most solvents, disinfectants, lipids, and bleaches [30,31]. It was also important that PP is a material used, among others, for medical devices, i.e., drug dispensers, Luer elements, connectors, syringes, and other small laboratory equipment [30]. Another important factor for choosing this material was the cost of production [32], fire resistance, high deformation temperature, dimensional stability, and recyclability [30].

It is well known that a low temperature aids in maintaining the integrity of RNA samples during storage and transportation [33,34]. Although freezing at −80 °C is the gold standard method for RNA storage [33,35,36,37], maintaining this condition during sample transportation increases the cost of this service. Moreover, as it was mentioned above, international regulations for the transport of infectious material are directly described by WHO [18]. Briefly, the regulations state that this kind of material must be transported in a triple packaging system that consists of (i) a leak-proof primary receptacle, (ii) leak-proof secondary packaging, and (iii) adequate outer packaging [18]. The use of the presented tool for storing or transporting human RNA samples allows for maintaining a lower temperature of up to 4 °C for no less than 45 min and no higher than 10–15 °C for approximately 2 h. Additionally, the original design of the container gives the possibility to change the volume of the coolant and prepare it to the user’s needs. The ergonomic shapes of the container are also useful for storing it in a laboratory refrigerator or freezer, including low-temperature ones. In this case, not only are the ability to maintain a low temperature and ergonomic features important for laboratory practice but also its biological safety.

The presented novel container ensures a maintained lowered temperature for several hours, making it useful in sustaining the conditions for transporting biological samples. It is also important that our container meets legal requirements for the transportation of infectious material. The lack of changes in our RNA sample integrity, purity, and concentration, with regards to spectroscopic, automated electrophoresis, RT-dPCR, and RT-qPCR data, are in line with the results found using an RNA shell ensuring the safe storage of nucleic acids at room temperature for long periods [35]. The stability of total RNA found in this study is in line with well-established knowledge that a controlled (anhydrous and anoxic) atmosphere can protect the nucleic acids from deleterious factors (air, water, and light) and allow for the preservation of nucleic acids at room temperature for longer periods [3,34,35]. The most important factor that influences the stability of total RNA is the presence and activity of ribonucleases (RNases). However, it was evidenced that RNA is more resistant to oxidative stress than DNA [34] and this might explain why there were no significant changes in the total RNA concentration in the liquid samples as compared to those stored without the cooled container (control probes) and those kept in it (test probes), excluding the 300 min time point. According to Brisco and Morley, RNA molecules in solution undergo between 0.7 and 7.6 cuts per 1000 nucleotides per 30 min at 90 °C [38]. On the other hand, the variety of local RNA conformation is generally responsible for heterogeneous RNA degradation rates [39]. It could also explain the results found in this study. The RNA degradation mechanism is described as spontaneous cleavage of the phosphodiester linkage through transesterification resulting from a nucleophilic attack of the phosphorus atom by the neighboring 2′OH group [3]. The reaction is highly dependent on the geometry of the molecule [39,40]. There are literature data evidencing that the dehydration of RNA strongly inhibits its degradation, and partial rehydration by atmospheric water restores the initial instability while still in the solid state [3,41]. On the other hand, at room-temperature conditions, the activation energy of the strand-breaking reaction is 28.5 kcal/mol. This value is close to the activation energy of the base-catalyzed cleavage in the solution of a 22-nucleotide-long RNA molecule [42], suggesting that the mechanism of RNA degradation in solution is similar to the model study and that lowering the molecular mobility may not be involved in the inhibition of such degradation. Taking this into account, it seems that changes in total RNA concentration in liquid samples are not necessary observations related to the quality of RNA matrices for future gene expression analyses in isolated probes.

Both RT-qPCR and RT-dPCR analyses revealed significant differences in the expression of the studied housekeeping genes when comparing control and test probes. Brisco and Morley enumerate three sources of RNA degradation during the RT-qPCR process, namely, “prior degradation of the RNA in the sample being assayed, degradation of RNA by RT or early termination of cDNA synthesis and degradation of the cDNA” causing differences between the original RNA molecules and the number of cDNA particles quantified by qPCR [38]. According to the literature data, short amplicons (70–250 bp) are more or less independent of a slight degradation of the RNA quality, whereas products exceeding 400 bp are strongly dependent [3,43]. There are data evidencing that the degradation of total RNA could raise the efficiency of the reverse transcription reaction [44,45] probably due to suppression of the inhibitory effect of the RNA secondary structure [46]. On the other hand, RNA degradation affecting the final results of an RT-qPCR analysis may be the result of RNase activity in RT. Moreover, unlike traditional RT-qPCR, RT-dPCR provides a linear response to the number of copies present in the sample to allow for small-fold change differences to be detected. RT-dPCR, by the means of (1) the partitioning of the PCR reaction into thousands of individual reaction vessels before amplification and (2) the acquisition of data at the reaction endpoint, gives more precise and reproducible data versus RT-qPCR especially in the presence of sample contaminants that can partially inhibit Taq polymerase and/or primer annealing [47]. This is a possible explanation for the differences found between the RT-qPCR and RT-dPCR data in the presented results. The analysis of RNA integrity explained the changes between the control and test probes during the incubation time. It is worth noting that during the incubation time, the samples from the control probe were incubated at room temperature without any RNA protection means resulting in decreasing its quality that was further reflected in the results of the RT-qPCR and RT-dPCR analyses.

Taking the sensitivity of diagnostic procedures into account, it is important to verify any novel procedures or novel pre-analytic tools using the most sensitive methods. This is why the RT-dPCR technique was chosen for the validation of our container for RNA sample transportation. Such a technique helps to reduce the level of biological material needed to perform molecular analysis [20,48] but requires more control over pre-analytical procedures. The higher levels of housekeeping gene cDNA found in samples kept in our container provide evidence that this container can help to avoid pre-analytical errors related to the degradation of nucleic acid during transportation.

Each study has its limitations. In the case of our research, using more housekeeping genes would reinforce the obtained data. It is planned to be performed in a future study.

## 4. Materials and Methods

### 4.1. Container Construction

**Figure 5:**
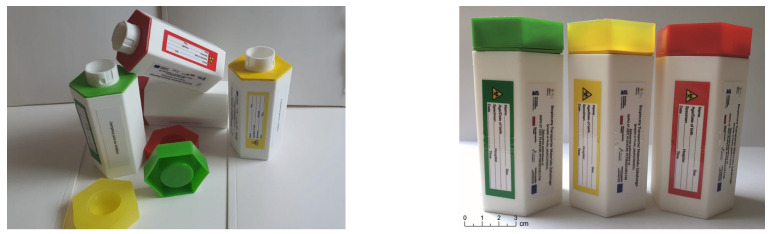
The container for secured transport of biological material.

The container for secured transport of biological material was designed in our laboratory and produced by injection molding technology using three types of material: polypropylene (PP) for the main body of the container; thermoplastic polyurethane elastomers (TPUs) for the gasket; and polyethylene (PE) for the nut (Figure 5). There are six chambers within the main body of the container, each filled with an absorbing substance and a cooling gel. It is a container for the safe transport of tubes with biological material, comprising an opening at a first end, a double-walled container body, and a closing means couplable with the body and adapted to seal the container at the first end. The container body has an inner circumferential wall and, surrounding it, an outer circumferential wall, wherein both the inner and outer walls extend coaxially. At the second end of the body, the inner wall is tightly joined peripherally with the bottom wall of the body, forming a transport chamber that is open at the first end of the body and intended to receive a tube with biological material. The outer wall of the container at the second end of the body is also tightly joined peripherally to the bottom wall and is also tightly joined along its entire extension between the first and second ends of the body to said inner and bottom walls, utilizing at least two partition walls. Thus, it forms at least two insulating chambers, sealed one from another by a partition wall. The insulating chambers surround said transport chamber arranged between them and the insulating chambers are open at the first end of the container body. At its first end, the body is provided with coupling means adapted to engage corresponding coupling means provided in said closing means of the container. When engaged, said coupling means tightly closes said transport chamber and preferably also said insulating chambers. The total volume of the transporting part of the container equals 49.8 cm^3^. The general construction, dimensions, and 3D image of the transporter are presented in Supplementary Figure S3. More information about the technical construction of the container is presented in the utility model pending application: No. W.132412 [WIPO ST 10/C PL132412U].

The containers were tested for mechanical and temperature resistance. In order to determine the tightness of the container, pieces of Whatman filter paper (in the shape of rectangles, 2 × 6 cm) were closed inside the container, which was placed for approximately 12 h at −20 °C. The coolant used in the container was colored with a 0.01% solution of methylene blue and so, in the event of a leak, the Whatman paper would absorb the solution and turn blue. The tests were performed in triplicate for 100 containers.

To determine the internal temperature profile of the container, they were placed for 12 h at −20 °C for pre-cooling. The containers were then moved to room temperature, where a temperature probe from the MEATER Block (Apption Labs, Leicester, UK) set was immediately placed inside each container. The data of the internal and ambient temperature sent by the probes were collected by a dedicated smartphone application (MEATER for Android, v2.11.0, Apption Labs, Leicester, UK). The observation was carried out until reaching room temperature (25 °C). The tests were performed in triplicate for 100 containers.

### 4.2. Analysis of RNA Stability in the Container

#### 4.2.1. Isolation of RNA from Peripheral Nuclear Blood Cells

The total RNA was isolated from peripheral blood leukocyte samples using the GeneMatrix Human Blood RNA Purification Kit (EURx, Gdańsk, Poland) according to the manufacturer’s protocol. Potential genomic DNA contamination was eliminated by DNase I (Thermo Fisher Scientific, Waltham, MA, USA) treatment of all the RNA samples. The RNA concentration and purity of each sample were evaluated using a NanoDrop™ 2000 Spectrophotometer (Thermo Fisher Scientific, Waltham, MA, USA) and the data are presented in Supplementary Table S1.

All the testing procedures, including those requiring the donation of volunteers’ blood samples approved by the Local Ethics Committee at the Regional Medical Chamber in Szczecin (approval no. 05/KB/VII/2019), were approved by the “Socially responsible Proto_lab” project board under the West Pomeranian Voivodeship and carried out in accordance with the relevant guidelines and regulations. The participants were fully informed of the experimental procedures before giving their written informed consent to participate.

#### 4.2.2. RNA Integrity Measurement

The RNA quality of the samples, which were isolated from peripheral blood leukocytes, were assessed using the RIN value obtained from the TapeStation System (Agilent Technologies, Inc., Santa Clara, CA, USA). The RIN values were performed in each time point according to the manufacturer’s protocol and the experiment was performed in triplicate.

#### 4.2.3. Reverse Transcription

The single-stranded cDNA of each sample was synthesized from 1 μL total RNA in a 20 μL reaction volume using the RevertAid Reverse Transcription Kit (Thermo Fisher Scientific, Waltham, MA, USA) according to the manufacturer’s protocol. The obtained cDNA samples were stored at −20 °C for further RT-qPCR and RT-dPCR analyses. The reverse transcription of RNA in both the control and studied probes were performed immediately after the end of the incubation time.

#### 4.2.4. qPCR Protocol

The amplification of selected genes from the cDNA template was performed by RT-qPCR using PowerUp™ SYBR™ Green Master Mix (Thermo Fisher Scientific, Waltham, MA, USA) with the use of the CFX96 Touch Real-Time PCR System (Bio-Rad, Hercules, CA, USA). The primers used in the RT-qPCR analyses are listed in Table 2. The reaction mixture contained PowerUp™ SYBR Green Master Mix, 200 nM of reverse and forward primers, and template cDNA diluted in the same way as for the RT-dPCR protocol (see below) constituting 1/10 of the total mixture volume. The cycling conditions (temperature and time) were determined according to the manufacturer’s instructions, taking into consideration the melting temperatures of the primers. Amplification was initiated at 50 °C for 2 min, next at 95 °C for 2 min, followed by 40 cycles of 95 °C for 15 s and 55–60 °C for 15 s and the extension at 72 °C for 1 min. Additionally, to exclude nonspecific products, the melting curves of the PCR products were analyzed after termination of the amplification reaction. The obtained reaction products from each pair of primers were sequenced by an external company (Genomed S.A., Warsaw, Poland) to confirm the result (Supplementary Figures S4–S6). Additionally, the dilution curves for the target genes’ RT-qPCR analyses are provided in Supplementary Figure S7.

#### 4.2.5. RT-dPCR Protocol

The expression levels of the selected genes were quantified by RT-dPCR using the QIAcuity EG PCR Kit (Qiagen, Hilden, Germany) on a QIAcuity One 5plex Digital PCR Detection System (Qiagen, Hilden, Germany). The same primers as in the RT-qPCR protocol were used (Table 2). The reaction mixture was prepared by combining 4 µL EvaGreen PCR Master Mix (3×) (Qiagen, Hilden, Germany) and 400 nM of reverse and forward primers. For the highest efficiency in RT-dPCR, similarly to RT-qPCR, the amplicons were shorter than 300 bp in length. The volume of the cDNA added was not higher than 10% of the total reaction volume. Reaction validation was performed in RT-qPCR in terms of the primer concentrations and annealing temperature, and they were also used for the RT-dPCR analyses. As a result, 1.2 microliters of an appropriately diluted nucleic acid sample (*ACTB* 10×, *GAPDH* 100×, and *Rack1* 100×, respectively) was added to the amplification mixture, which contained 12 μL of each reaction system. The reaction was carried out on a QIAcuity Nanoplate 8.5k 24-well (Qiagen, Hilden, Germany). Amplification was initiated at 95 °C for 2 min, followed by 40 cycles of 95 °C for 15 s and 58 °C for 60 s and the final extension at 98 °C for 10 min.

### 4.3. Statistical Analysis

All the data are presented as the mean ±SD. The statistical analyses were carried out using Statistica (version 13, TIBCO Software Inc., Palo Alto, CA, USA, 2017). The normality of the data distribution within the subgroups was assessed using the Shapiro–Wilk test. The differences between the control and test values were analyzed using the *t*-test for independent samples, while a comparison of the time points in each group (control and test) was performed using the analysis of variance (ANOVA) with repeated measures. In the case of significant ANOVA results, a post hoc analysis using the Tukey HSD test or Tukey Unequal N HSD test was performed. For all the analyses, a *p* value < 0.05 was considered significant.

## 5. Conclusions

The novel container presented in this study effectively stores and transports biological material. It helps to retain the quality of total RNA over time so that RNA samples can be used to accurately analyze biological material that has been transported for several hours.

## 6. Patents

Technical construction of the container is subject of the utility model pending application: No. W.132412 [WIPO ST 10/C PL132412U].

## Figures and Tables

**Table 1:** RNA integrity of studied samples.

T [min]	Control Probe [RIN]	Test Probe [RIN]	p_t_^1^
0	9.6 ± 0.1	9.7 ± 0.1	0.600
15	9.5 ± 0.2	9.6 ± 0.1	0.534
30	9.5 ± 0.2	9.7 ± 0.1	0.051
60	9.4 ± 0.1	9.6 ± 0.1	0.003
90	8.3 ± 0.6 ***	9.4 ± 0.2 ***	0.001
120	8.3 ± 0.4 ***	9.5 ± 0.1	<0.001
180	8.7 ± 0.2 ***	9.6 ± 0.1	<0.001
240	7.8 ± 0.7 ***	9.5 ± 0.1	<0.001
300	7.9 ± 0.4 ***	9.6 ± 0.1	<0.001

^1^ p_t_—*p* values for comparison control vs. test probe; *** *p* < 0.001, (compared to 0 min).

**Table 2:** qPCR primers used in this study.

Gene	Gene Name	Gene Function	Sequence (5′→3′)		Length	T_M_ ^1^ (°C)	T_A_ ^2^ (°C)	Amplicon Size (bp)	GenBank Accession Number
*ACTB*	Actin Beta	Cytoskeletal structural protein	Forward	CATGTACGTTGCTATCCAGGC	21	59.1	56	250	NM_001101
			Reverse	CTCCTTAATGTCACGCACGAT	21	58.4			
*Rack1*	Receptor for activated C kinase 1	Core ribosomal protein of the eukaryotic small (40S) ribosomal subunit	Forward	GATGTGGCCTTCTCCTCTG	20	59.8	60	224	NM_006098
			Reverse	GCTTGCATTAGCCAGGTTC	20	59.4			
*GAPDH*	Glyceraldehyde-3-phosphate dehydrogenase	Glycolysis pathway enzyme	Forward	GAAGGTGAAGGTCGGAGTC	19	57.2	60	226	NM_002046
			Reverse	GAAGATGGTGATGGGATTTC	20	53.7			

^1^ T_M_—melting temperature of the starters. ^2^ T_A_—annealing melting temperature used for the primers.

## Data Availability

The original contributions presented in this study are included in the article/Supplementary Material. Further inquiries can be directed to the corresponding author.

## Supplementary Materials

The following supporting information can be downloaded at: https://www.mdpi.com/article/10.3390/ijms26010228/s1.
