# Supplementary material for: The Use of a Novel Container for Secured Transport and Storage of Biological Material for Quantitative Human RNA Analysis
Source: Int J Mol Sci. 2024 Dec 30;26(1):228. doi: 10.3390/ijms26010228 (PMC11719513; doi:10.3390/ijms26010228)
Supplement: Supplementary file 1 [file ijms-26-00228-s001.zip › ijms-3357486-revised-supplementary-material.pdf]

Supplementary material

The use of a novel container for secured transport and storage of biological material for quantitative human RNA analysis

Dorota Kostrzewa-Nowak, Alicja Trzeciak-Ryczek, Klaudyna Lewandowska, Thierry van de Wetering, Andrzej Ciechanowicz and Robert Nowak *

***** Correspondence: robert.nowak@usz.edu.pl

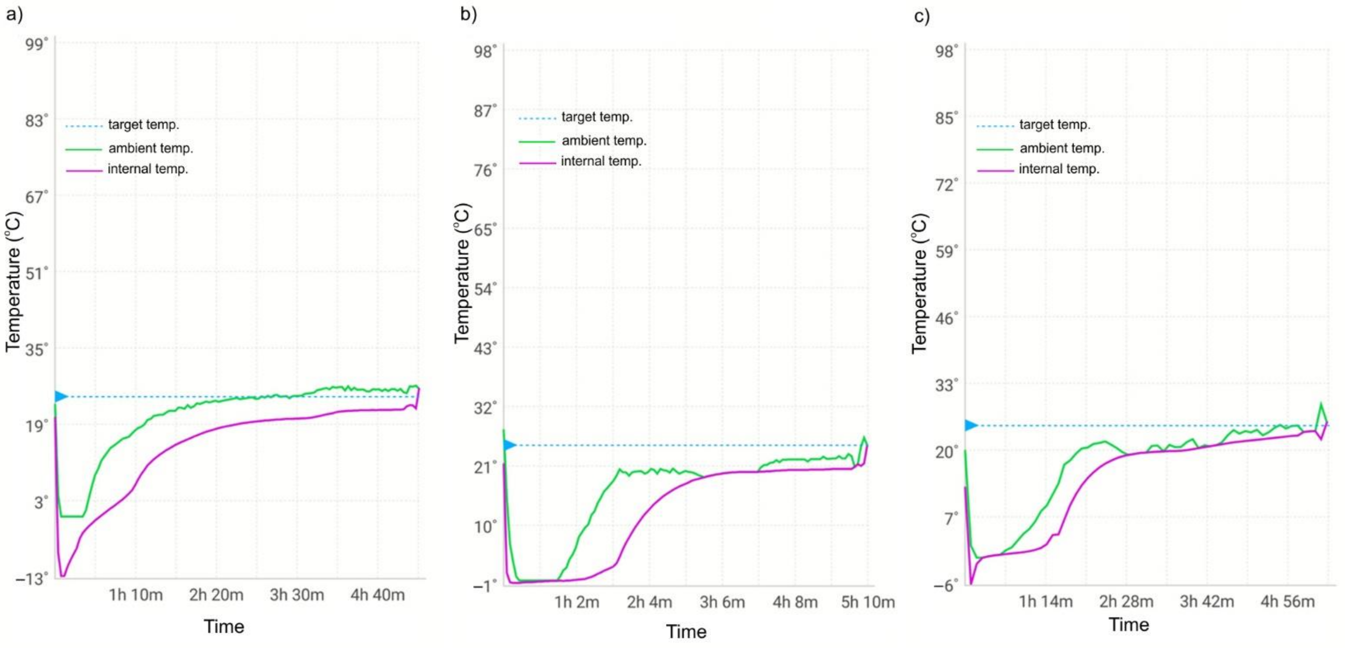

Figure S1. Representative examples of temperature profiles of the containers 3D printed using a) ABS, b) PETG, c) PLA. The blue dotted line indicates the target temperature value of +25°C.

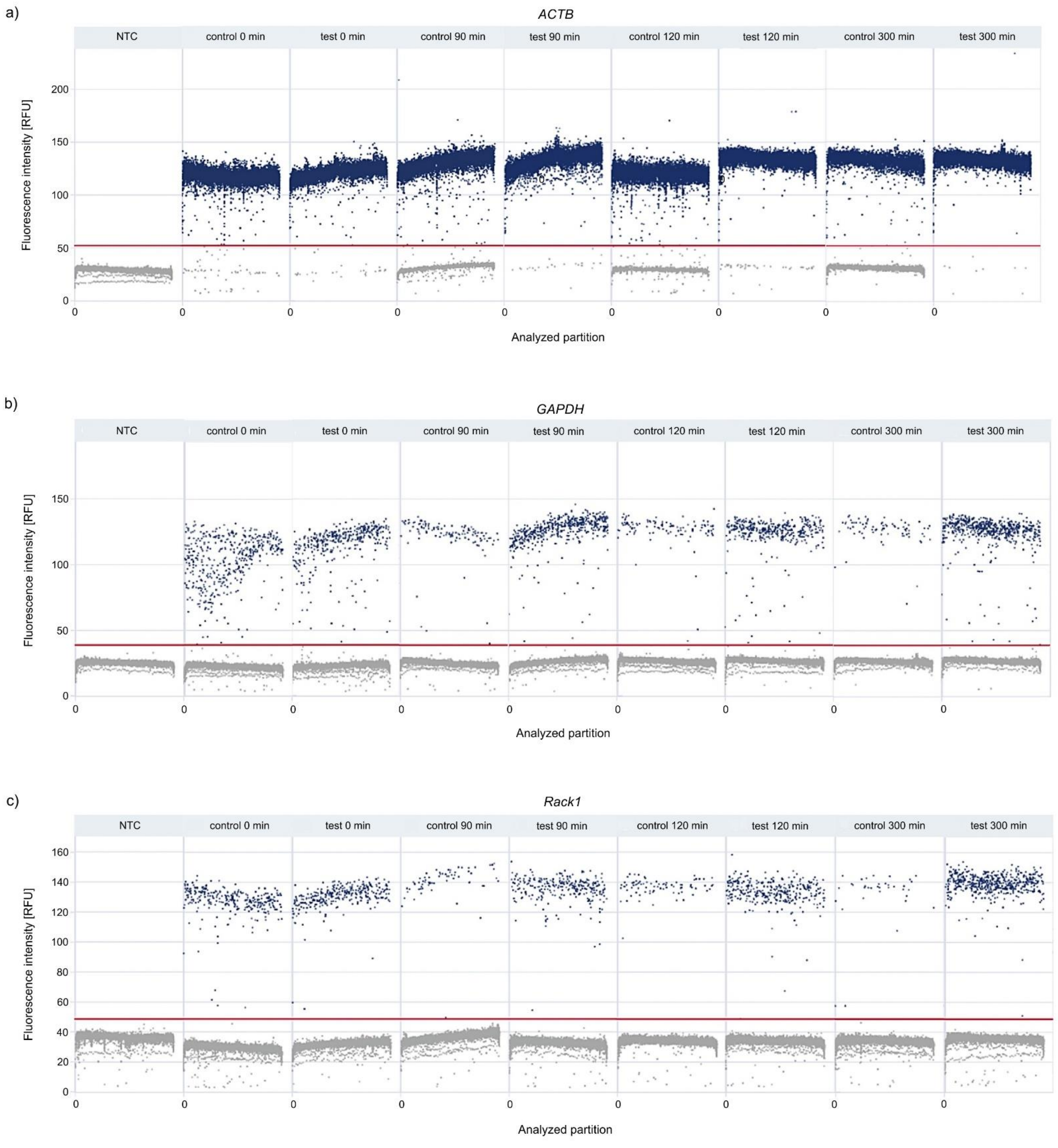

Figure S2. Representative results of the dPCR droplets of a) *ACTB*, b) *GAPDH*, c) *Rack1* genes.

a)

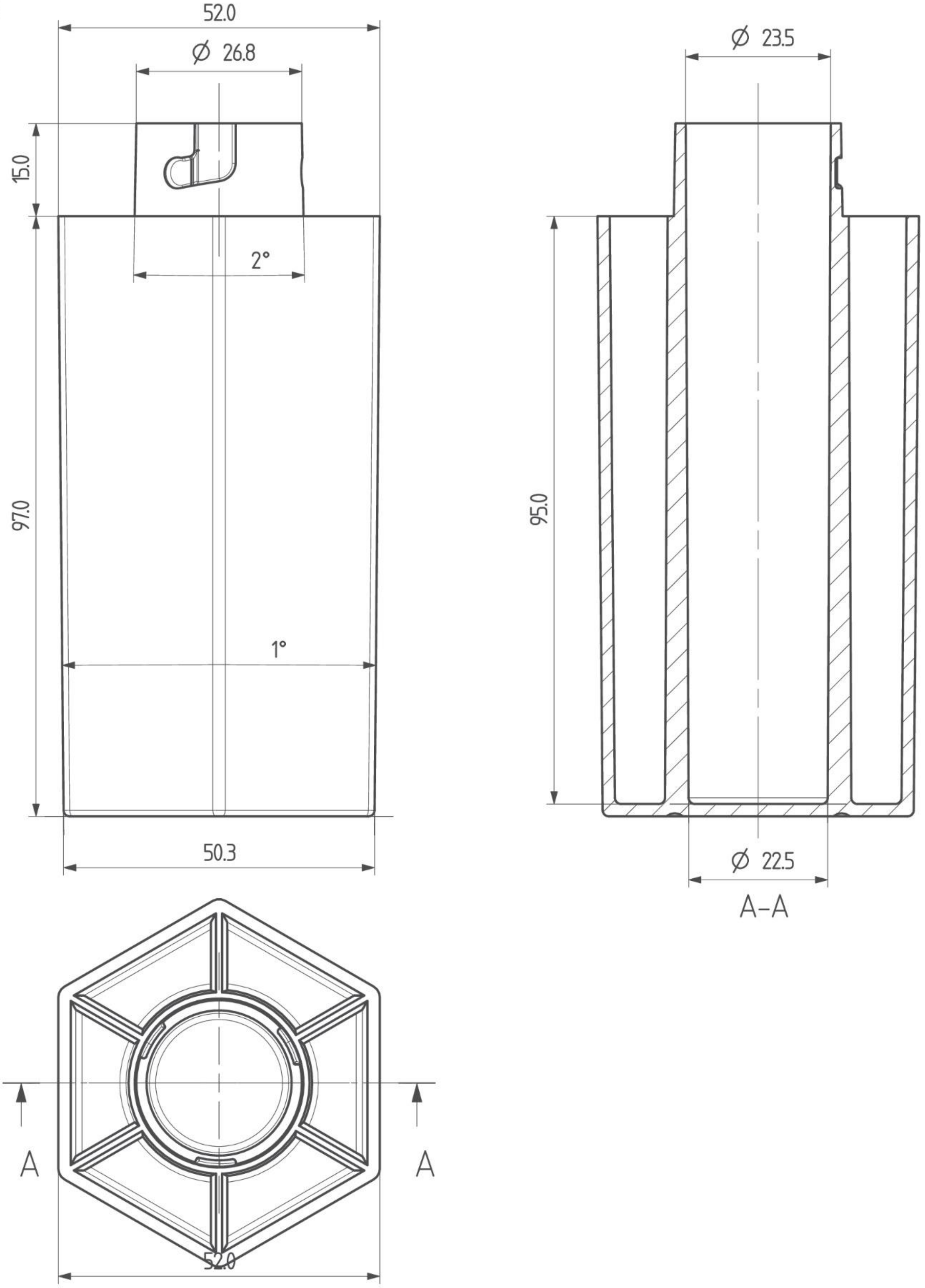

b)

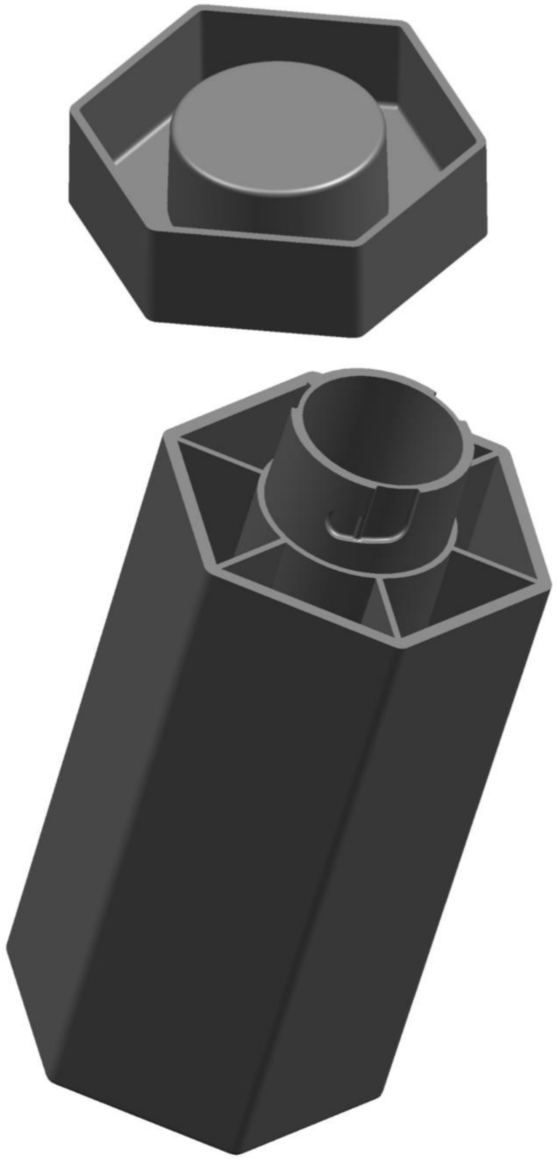

Figure S3. a) General construction, dimensions and b) 3D image of the container for the secured transport of biological material.

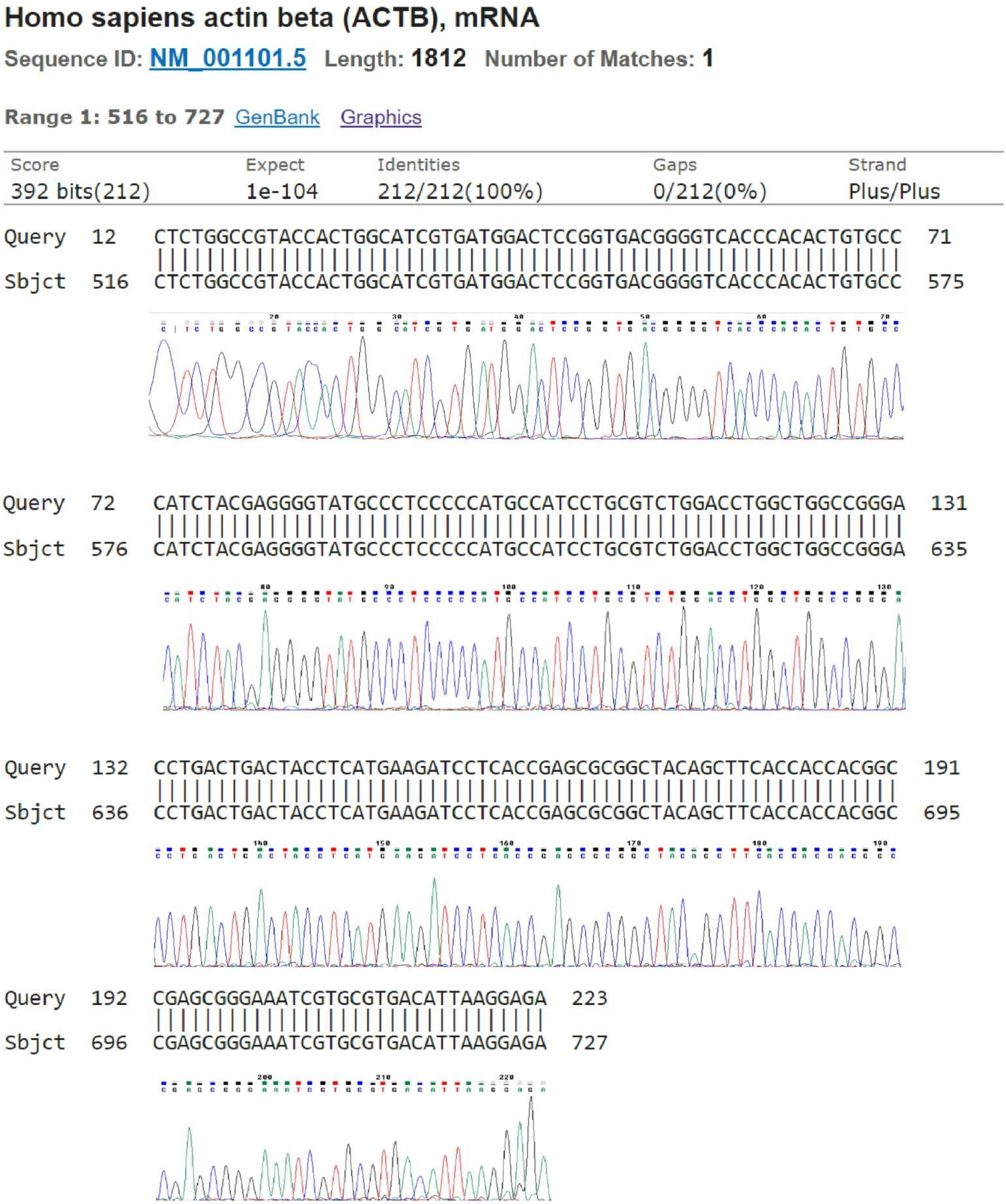

Figure S4. The alignment of ACTB amplicon to the *ACTB* reference sequence. The sequences were verified using Basic Local Alignment Search Tool (BLAST®; available at https://blast.ncbi.nlm.nih.gov/Blast.cgi). Chromatograms were generated using Chromas ver. 2.6.6 (Technelysium Pty Ltd., South Brisbane, Australia; available at http://technelysium.com.au/wp/chromas/).

Homo sapiens glyceraldehyde-3-phosphate dehydrogenase (GAPDH), transcript variant 1, mRNA

Sequence ID: NM_002046.7 Length: 1285 Number of Matches: 1

Range 1: 116 to 309 GenBank Graphics

Score	Expect	Identities	Gaps	Strand
359 bits(194)	9e-95	194/194(100%)	0/194(0%)	Plus/Plus

```
Query  10   ATTGGGCGCCTGGTCACCAGGGCTGCTTTTAACTCTGGTAAAGTGGATATTGTTGCCATC  69
            ||||||||||||||||||||||||||||||||||||||||||||||||||||||||||||
Sbjct  116  ATTGGGCGCCTGGTCACCAGGGCTGCTTTTAACTCTGGTAAAGTGGATATTGTTGCCATC  175
```

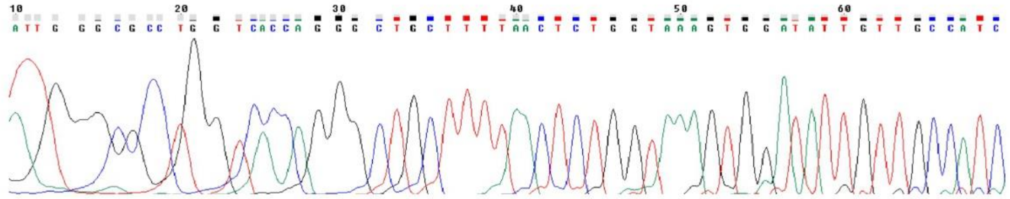

```
Query  70   AATGACCCCTTCATTGACCTCAACTACATGGTTTACATGTTCCAATATGATTCCACCCAT  129
            ||||||||||||||||||||||||||||||||||||||||||||||||||||||||||||
Sbjct  176  AATGACCCCTTCATTGACCTCAACTACATGGTTTACATGTTCCAATATGATTCCACCCAT  235
```

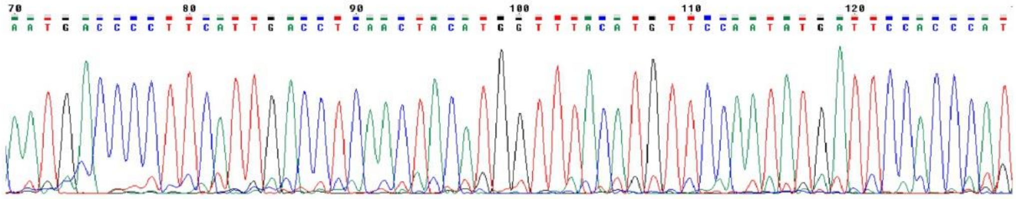

```
Query  130  GGCAAATTCCATGGCACCGTCAAGGCTGAGAACGGGAAGCTTGTCATCAATGGAAATCCC  189
            ||||||||||||||||||||||||||||||||||||||||||||||||||||||||||||
Sbjct  236  GGCAAATTCCATGGCACCGTCAAGGCTGAGAACGGGAAGCTTGTCATCAATGGAAATCCC  295
```

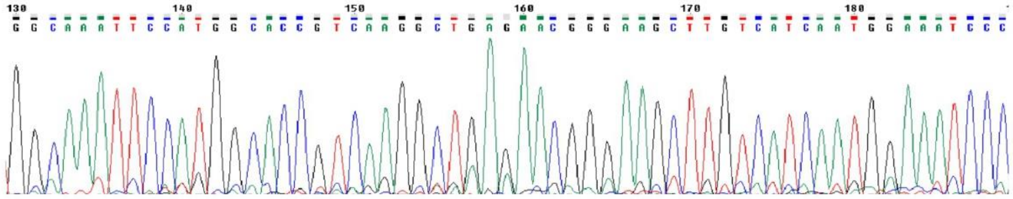

```
Query  190  ATCACCATCTTCCA  203
            ||||||||||||||
Sbjct  296  ATCACCATCTTCCA  309
```

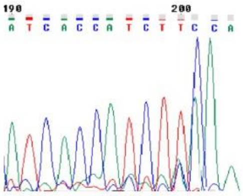

Figure S5. The alignment of *GAPDH* amplicon to the *GAPDH* reference sequence. For details, see legend for Figure S4.

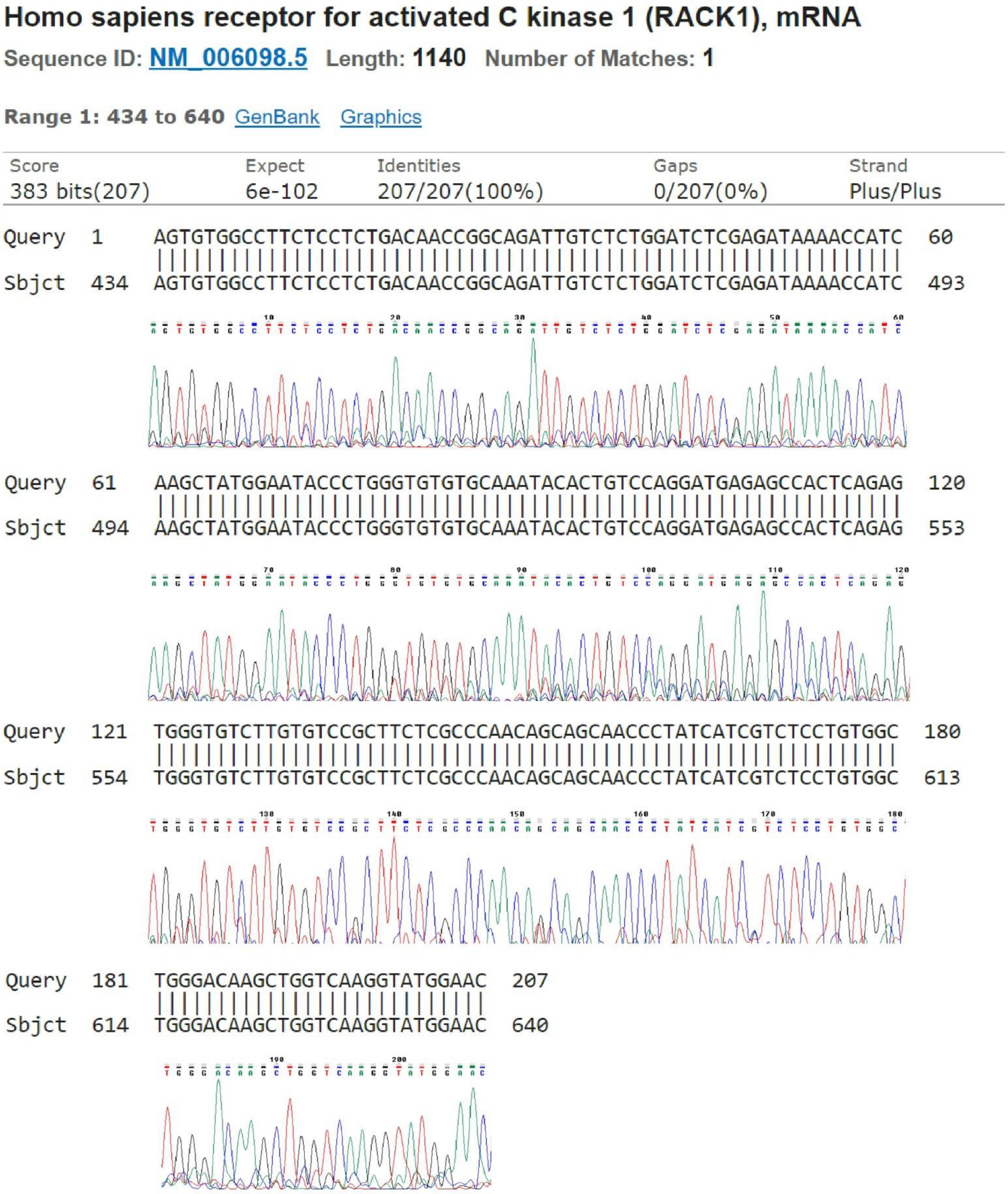

Figure S6. The alignment of *Rack1* amplicon to the *Rack1* reference sequence. For details, see legend for Figure S4.

a)

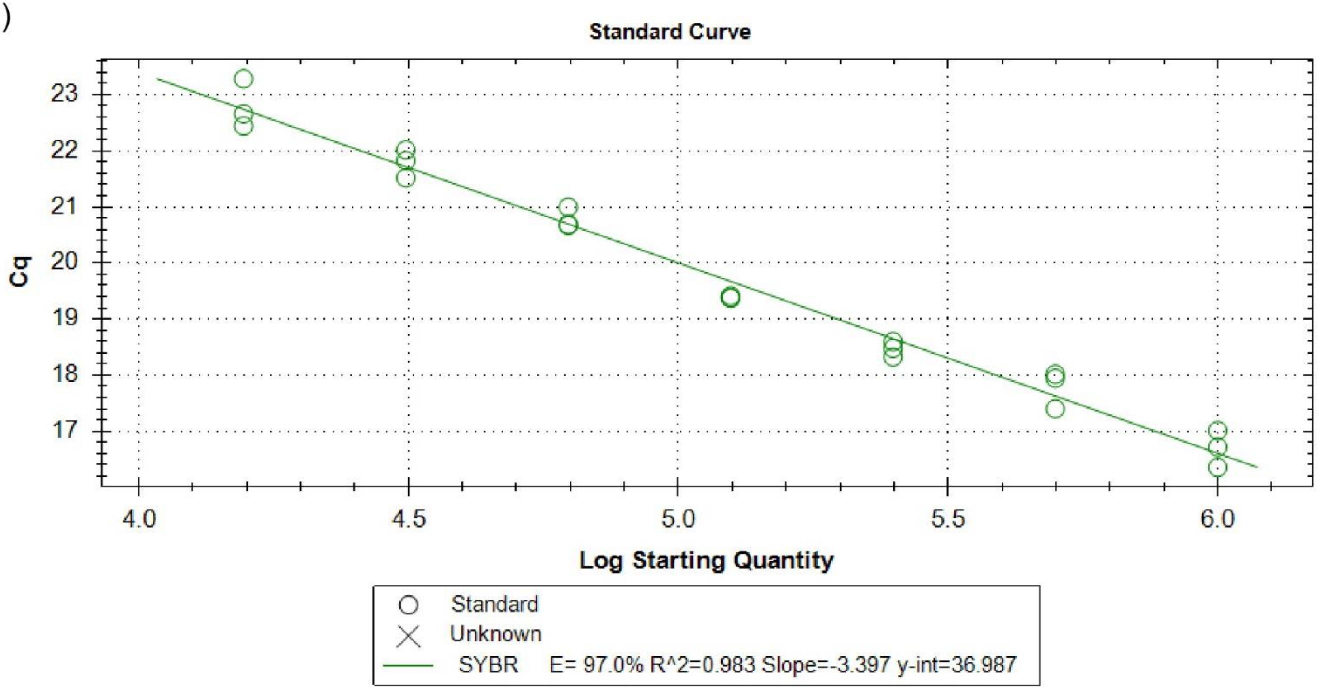
Standard Curve
23
22
21
20
19
18
17
Cq
4.0
4.5
5.0
5.5
6.0
Log Starting Quantity
Standard
Unknown
SYBR E= 97.0% R^2=0.983 Slope=-3.397 y-int=36.987

b)

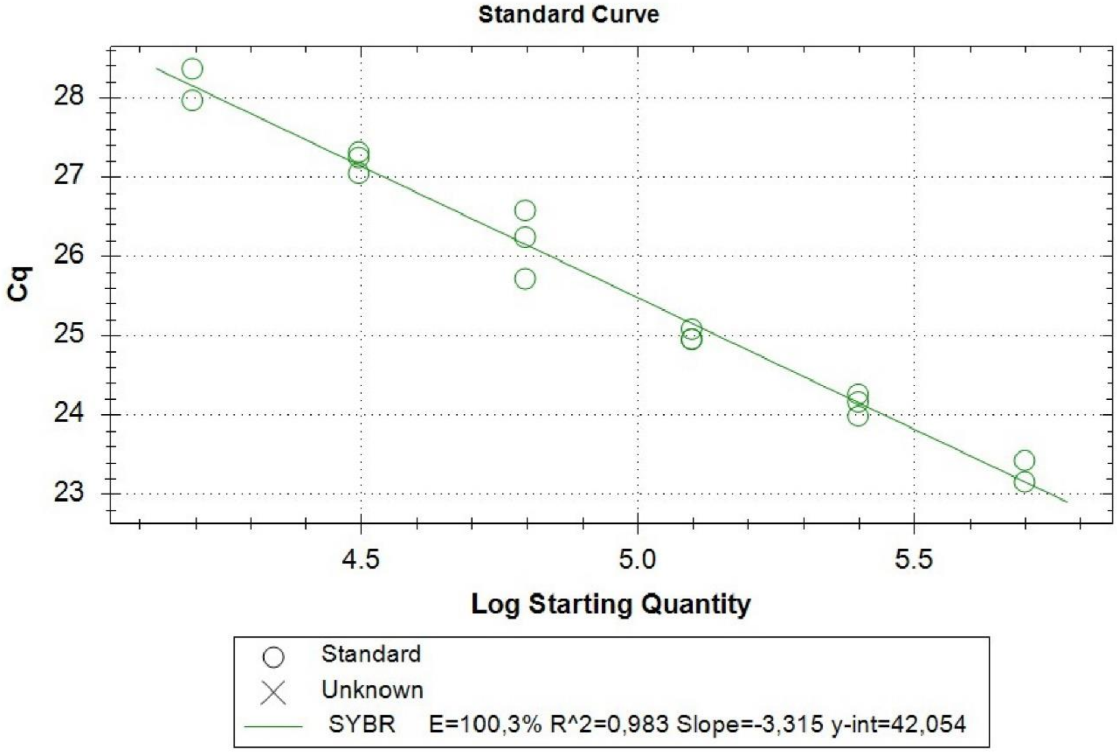
Standard Curve
28
27
26
25
24
23
Cq
4.5
5.0
5.5
Log Starting Quantity
Standard
Unknown
SYBR E=100,3% R^2=0,983 Slope=-3,315 y-int=42,054

c)

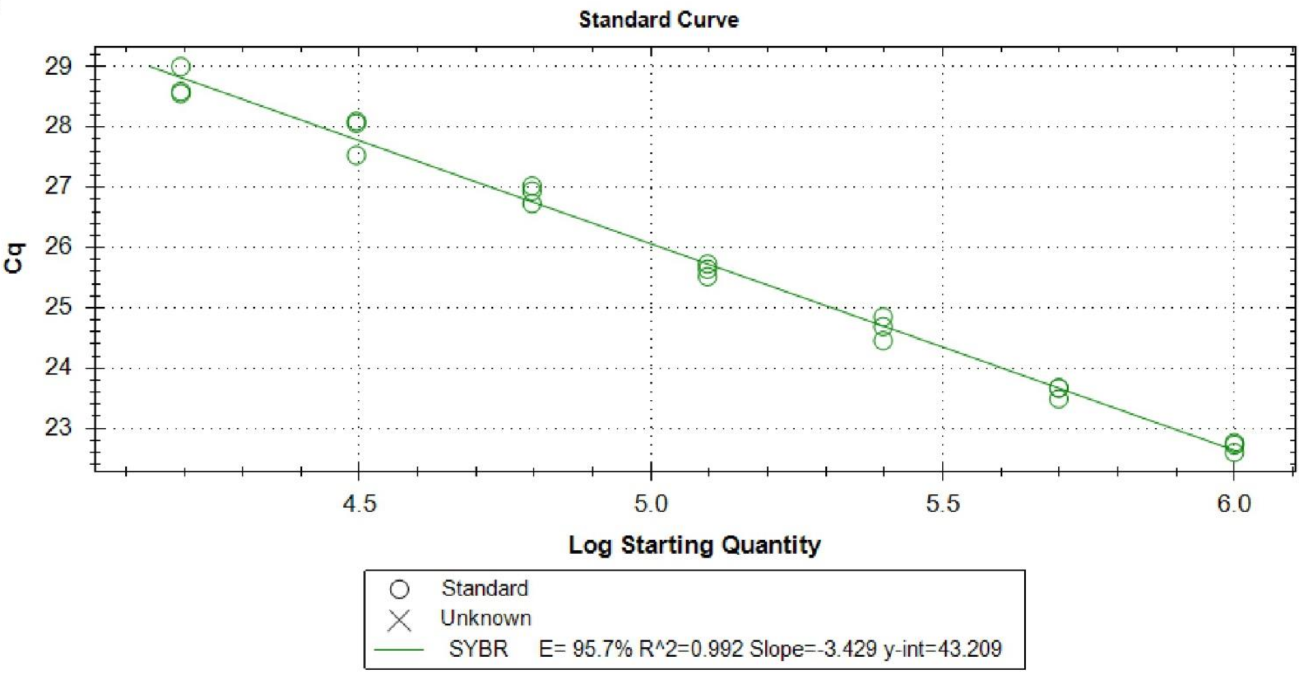

Figure S7. Dilution curves for a) *ACTB*, b) *GAPDH*, c) *Rack1* genes' RT-qPCR analyses.

Table S1. The concentration (C) and purity of total RNA in control and test probes measured using spectroscopic method.

**T [min]**	**Control probe**			**Test probe**		
	**C (ng/µL)**	**A_{260}/A_{280}**	**A_{260}/A_{230}**	**C [ng/µL]**	**A_{260}/A_{280}**	**A_{260}/A_{230}**
0	42.10 ±0.53	2.20 ± 0.03	0.80 ± 0.03	40.80 ± 0.80	2.20 ±0.05	0.90 ± 0.04
15	41.80 ± 0.48	2.16 ± 0.04	0.88 ±0.04	41.45 ± 0.44	2.18 ± 0.05	0.89 ±0.02
30	42.44 ± 0.56	2.16 ± 0.03	0.89 ± 0.03	43.28 ± 2.25	2.18 ± 0.02	0.91 ± 0.02
60	41.91 ± 1.08	2.15 ± 0.05	0.81 ± 0.09	41.12 ± 0.75	2.17 ± 0.03	0.88 ± 0.01
90	43.00 ± 0.50	2.10 ± 0.05	0.80 ± 0.03	42.40 ± 0.75	2.10 ± 0.04	0.80 ± 0.05
120	42.40 ± 0.52	2.10 ± 0.05	0.90 ± 0.03	41.60± 1.54	2.10 ± 0.04	0.90 ± 0.06
180	43.00 ± 0.74	2.21 ± 0.29	0.79 ± 0.12	44.17 ± 1.55	2.13 ± 0.05	0.84 ± 0.01
240	42.83 ± 0.73	2.11 ± 0.10	0.79 ± 0.09	44.38 ± 2.19	2.14 ± 0.07	0.84 ± 0.02
300	41.51 ± 0.52	2.17 ± 0.05	0.82 ± 0.13	44.10 ± 1.62	2.20 ± 0.03	0.90 ± 0.03
